# Changes in Cardiometabolic Risk Factors and Ischaemic Heart Disease Mortality Between 2000 and 2015: A Global Ecological Analysis

**DOI:** 10.3390/medicina62040617

**Published:** 2026-03-24

**Authors:** Hyemi Lee, Jang-Hun Jeong, Sang-Chul Kim, Yong-Dae Kim, Sang-Yong Eom

**Affiliations:** 1 Department of Preventive Medicine, College of Medicine, Chungbuk National University, Cheongju 28644, Republic of Koreamsf.jhj@cbu.ac.kr (J.-H.J.);; 2 Department of Emergency Medicine, College of Medicine, Chungbuk National University, Cheongju 28644, Republic of Korea; 3 Department of Emergency Medicine, Chungbuk National University Hospital, Cheongju 28644, Republic of Korea; 4 Chungbuk Environmental Health Center, Chungbuk National University Hospital, Cheongju 28644, Republic of Korea

**Keywords:** cardiometabolic risk factors, cholesterol, ischaemic heart disease, age-standardized death rate, ecological study

## Abstract

*Background and Objectives*: To assess whether country-level changes in major cardiometabolic risk factors were associated with concurrent changes in age-standardized death rates (ASDR) from ischaemic heart disease (IHD) and stroke between 2000 and 2015. *Materials and Methods*: We conducted a multinational ecological analysis using harmonized data from WHO, NCD-RisC, and the World Bank across 157 countries (n = 157). Absolute changes in systolic blood pressure, obesity, diabetes, and total cholesterol were standardized to z-scores. Linear regression models examined change–change associations, adjusting for income group and behavioral factors. Income-stratified and quartile-based analyses were performed. *Results*: Between 2000 and 2015, IHD and stroke mortality declined, while obesity and diabetes increased. In adjusted models, change in total cholesterol showed the most consistent association with change in IHD ASDR (β = 13.09, 95% CIs = 4.58–21.60, *p* = 0.003), whereas the other risk factors did not show consistent independent associations. Significant associations were confined to high- and upper middle-income countries, where change in total cholesterol was associated with IHD mortality. *Conclusions*: Changes in total cholesterol showed the most consistent correspondence with concurrent changes in IHD mortality at the country level, whereas other cardiometabolic risk factors showed less consistent patterns. These patterns were observed mainly in high- and upper–middle-income countries, suggesting heterogeneity by socioeconomic context.

## 1. Introduction

Ischaemic heart disease (IHD) and ischaemic stroke (IS) remain the leading causes of death worldwide, accounting for a substantial share of the global disease burden and premature mortality. The World Health Organization (WHO) estimates that IHD and cerebrovascular disease together account for more than 20% of all deaths and a substantial proportion of the global disease burden in terms of disability-adjusted life-years (DALYs) [1]. Among these, IS represents a particularly large burden, with a global prevalence exceeding 104 million cases in 2017 and an annual incidence of approximately 12 million cases, making it a leading cause of death and disability in adults [2].

Metabolic risk factors account for most of the global cardiovascular disease (CVD) burden. In 2019, an estimated 13.7 million CVD deaths were attributable to metabolic factors, representing 73.7% of all CVD-related deaths worldwide [3]. Earlier global analyses have similarly shown that high blood pressure, high body mass index (BMI), elevated glucose, and high cholesterol are major contributors to mortality from CVD, chronic kidney disease, and diabetes: in 2010, high blood pressure alone accounted for over 40% of deaths from these conditions, while high BMI and glucose each contributed approximately 15%, and high cholesterol about 10%. After accounting for multi-causality, approximately 63% of deaths (10.8 million) from these diseases in 2010 were attributable to the combined effects of these four metabolic risk factors, highlighting their dominant role in driving the global CVD mortality burden [4].

These cardiometabolic risk factors frequently cluster as metabolic syndrome, characterized by abdominal obesity, elevated blood pressure, hyperglycemia, and dyslipidemia. The co-occurrence of these abnormalities confers greater cardiovascular risk than their independent effects [5]. The burden of CVD attributable to metabolic risk factors also varies substantially by socioeconomic development status, age, and sex across countries and regions [3].

To date, multinational studies examining metabolic risk factors and CVD burden have largely focused on describing the levels and temporal trends of mortality and disability, or on estimating the proportion of disease burden attributable to specific risk factors. For example, Global Burden of Disease–based analyses have quantified ASDR and DALYs attributable to hyperglycemia, elevated blood pressure, obesity, dyslipidemia, and impaired kidney function across regions, sociodemographic strata, and sex, primarily providing burden- and level-based descriptions of CVD, IHD, and stroke [3,4,6]. However, these studies have not directly evaluated whether countries experiencing greater worsening or improvement in cardiometabolic risk factors also exhibit corresponding changes in cardiovascular mortality over time, as their primary focus has been on attributable burden and cross-sectional or period-specific levels rather than on change–change associations [3,6]. Importantly, examining change–change relationships provides a complementary perspective to burden-based analyses by focusing on whether shifts in population risk factor profiles occur in parallel with changes in cardiovascular mortality. This approach may offer additional insight into how improvements or deteriorations in cardiometabolic risk factors are associated with population-level mortality trends across countries.

In addition, although some regional ecological studies have explored temporal associations between specific risk factor management indicators, such as population hypertension control, and cardiovascular mortality, these analyses have typically been restricted to particular regions or single risk factors and have relied on level-based associations between exposure and outcome [7]. From a policy perspective, evidence remains scarce from multinational analyses that systematically compare how much cardiometabolic risk factors have worsened or improved over the past two decades and how these changes have moved in parallel with changes in mortality from IHD and IS [3,4,6]. Few multinational studies have formally examined whether the associations between cardiometabolic risk factors and cardiovascular mortality vary according to national income level, despite frequent stratified descriptions of burden by sociodemographic development [3,6].

To address these gaps, we conducted a multinational country-level analysis to examine whether changes in major cardiometabolic risk factors are associated with concurrent changes in ASDR from IHD and IS. Using harmonized data from the WHO, the NCD Risk Factor Collaboration, and the World Bank, we quantified changes in blood pressure, obesity, diabetes, and total cholesterol between 2000 and 2015. We then assessed the association between these risk factor changes and corresponding changes in ASDR across countries. We further explored potential heterogeneity in these associations by national income level. The primary analysis focused on the period between 2000 and 2015, which represents the most recent interval with complete and comparable data available across all cardiometabolic risk factors.

By focusing on change–change relationships rather than level-based associations, this study aims to provide policy-relevant evidence on how shifts in cardiometabolic risk factor profiles at the population level translate into changes in cardiovascular mortality across countries.

## 2. Materials and Methods

### 2.1. Study Design and Data Source

We conducted a multinational, country-level ecological study to examine whether changes in major cardiometabolic risk factors were associated with concurrent changes in ASDR between 2000 and 2015. ASDR data were obtained from the WHO mortality database. Cause-specific outcomes were defined using the International Classification of Diseases (ICD) categories provided in the WHO dataset.

Country-level cardiometabolic risk factor estimates were obtained from the NCD Risk Factor Collaboration (NCD-RisC), including mean systolic blood pressure, obesity prevalence (BMI ≥ 30 kg/m^2^), diabetes prevalence, and mean total cholesterol. Data on behavioral indicators were obtained from the WHO Global Health Observatory, including prevalence of current tobacco use, alcohol consumption, and prevalence of insufficient physical activity. National income level was classified using the World Bank income group (fiscal year 2025 classification).

The main analytic period compared changes between 2000 and 2015 because these two years represented the most recent time points with consistent overlap across mortality, risk factor, behavioral, and income classification datasets, enabling harmonized cross-country comparisons.

National income level was classified according to the World Bank income group (fiscal year 2025 classification) and was treated as a time-invariant structural covariate in all regression models. Countries were included if data on mortality outcomes, cardiometabolic risk factors, and health behavior indicators were available for both 2000 and 2015.

The primary analytic period focused on changes between 2000 and 2015, as these years represented the most recent time points with complete and consistent overlap across mortality outcomes, cardiometabolic risk factors, behavioral indicators, and income classification datasets, enabling harmonized cross-country comparisons. Although later WHO ASDR outcome data were available for 2019, 2020, and 2021, exposure data were not consistently available across all four cardiometabolic risk factors for those years. Specifically, systolic blood pressure data were available only through 2015 and total cholesterol data through 2018, whereas obesity and diabetes extended further. As a result, analyses using later time points would necessarily have been limited to a subset of exposures and would not have been directly comparable with the main four-factor analytical framework.

### 2.2. Outcomes

We examined five cause-specific outcomes based on ASDR (deaths per 100,000 population): hypertensive heart disease (HHD), IHD, total stroke, IS, and haemorrhagic stroke (HS). For each country, ASDR were extracted for 2000 and 2015, and absolute changes were calculated as the difference between 2015 and 2000 (ΔASDR = ASDR_2015 − ASDR_2000). The main analysis used the “Persons” category to represent overall population mortality.

### 2.3. Cardiometabolic Risk Factors

We focused on four major cardiometabolic risk factors: systolic blood pressure, obesity prevalence (BMI ≥ 30 kg/m^2^), diabetes prevalence, and total cholesterol. For each country, absolute changes between 2000 and 2015 were computed for each risk factor (ΔX = X_2015 − X_2000). To enable direct comparison across risk factors measured on different scales, each change variable was standardized using z-score transformation across countries (Z = (ΔX − mean[ΔX])/SD[ΔX]). This standardization allows regression coefficients to be interpreted on a common scale, facilitating comparison of the relative strength of associations across risk factors. However, z-score normalization reflects relative differences across countries rather than absolute clinical changes, and therefore the resulting effect estimates should not be interpreted in terms of clinical magnitude or risk thresholds. In addition, standardization may obscure the underlying scale of change for individual risk factors. A composite metabolic change index was constructed by summing the four standardized risk-factor changes (Z-sum).

Risk factor estimates were harmonized at the population level using both-sex values. When sex-specific estimates were available in NCD-RisC, we averaged male and female estimates to derive a single both-sex value for each country-year to maximize comparability with the mortality and behavioral datasets.

### 2.4. Covariates

All regression models were adjusted for national income group (World Bank fiscal year 2025 classification) as a time-invariant structural covariate. National income level was included as a structural covariate because differences in socioeconomic development, healthcare access, and health system capacity may influence both population risk factor trends and cardiovascular mortality patterns.

Models were additionally adjusted for changes in behavioral indicators between 2000 and 2015, including change in tobacco use prevalence, change in alcohol consumption, and change in insufficient physical activity. These behavioral indicators were included as covariates because they represent major modifiable determinants of CVD and may confound the relationship between cardiometabolic risk factor trends and mortality changes at the population level.

Behavioral indicators were incorporated as both-sex measures to ensure consistency across countries and data sources. Absolute changes in these indicators between 2000 and 2015 were included in the regression models as continuous covariates to maintain consistency with the change–change analytical framework.

### 2.5. Statistical Analysis

**Figure 1:**
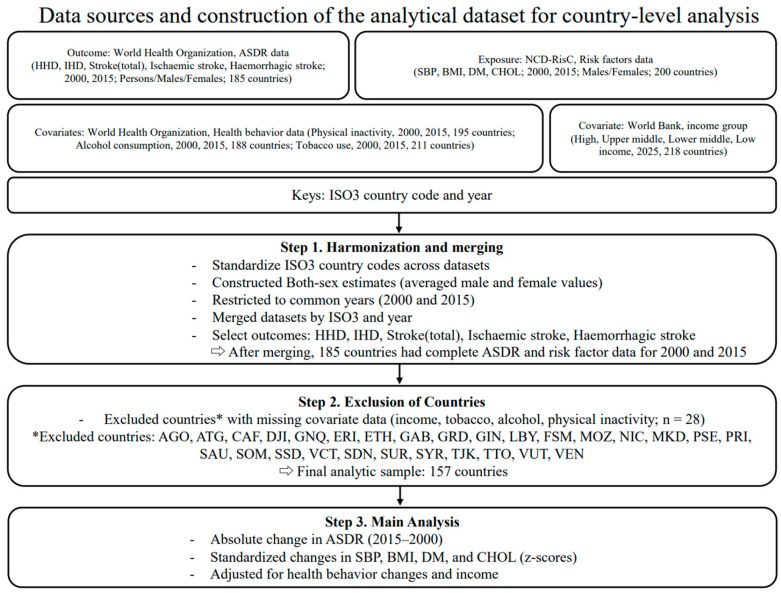
Flow diagram of data sources, merging, and construction of the analytic datasets.

All analyses were conducted at the country level. Mortality, cardiometabolic risk factor, behavioral, and income datasets were merged using ISO3 country codes. Countries with complete data for all mortality outcomes, cardiometabolic risk factors, behavioral covariates, and income classification in 2000 and 2015 were included in the primary analysis, resulting in a complete-case dataset of 157 countries (Figure 1).

Because all four cardiometabolic risk factors were concurrently available only in 2000 and 2015, the primary analysis employed a change–change design based on these two time points. For each country, absolute changes in ASDR (ΔASDR) and each cardiometabolic risk factor were calculated as 2015 minus 2000 values. Risk factor changes were standardized using z-score transformation to facilitate comparison across variables measured on different scales. A composite cardiometabolic change index (Z-sum) was constructed by summing the four standardized risk factor changes.

Associations between changes in risk factors and changes in mortality were examined using linear regression models with ΔASDR as the dependent variable. Separate models were fitted for HHD, IHD, total stroke, IS, and HS. Models were specified to evaluate individual standardized risk factor changes, all four standardized risk factor changes simultaneously, and the composite Z-sum index.

All adjusted models included national income group and absolute changes in smoking prevalence, alcohol consumption, and insufficient physical activity as covariates. In additional analyses, behavioral covariates were standardized using z-scores to assess robustness to differences in measurement scale. To evaluate model assumptions and potential multicollinearity, diagnostic checks were performed for the fully adjusted regression models. Multicollinearity among cardiometabolic risk factors and covariates was assessed using variance inflation factors (VIF). Linearity was evaluated through visual inspection of residual plots. Residual normality was evaluated using the Shapiro–Wilk test together with Q–Q plots, and heteroscedasticity was assessed using the Breusch–Pagan test. No substantial violations of model assumptions were identified. Sensitivity analyses were also conducted using alternative model specifications (e.g., non-standardized models). Effect modification by income level was explored through income-stratified analyses using the same model specifications.

Regression coefficients (β) with corresponding 95% confidence intervals (CIs) and *p*-values were reported. Statistical significance was defined using two-sided tests with an α level of 0.05. All analyses were conducted using R software (version 4.5.2; R Foundation for Statistical Computing, Vienna, Austria).

### 2.6. Ethics Statement

Ethical review and approval were not required for this study because it was an ecological analysis based exclusively on publicly available, open-access datasets. All data were fully de-identified and aggregated at the country level, and no individual-level information was accessible to the researchers. The study did not involve direct interaction with human participants, nor did it include any identifiable personal data. Therefore, the research did not meet the criteria for human subjects research requiring institutional ethical review.

## 3. Results

### 3.1. Study Population and Data Characteristics

Among countries with complete data on mortality outcomes, cardiometabolic risk factors, behavioral indicators, and income classification for both 2000 and 2015, a total of 157 countries were included in the primary analysis. Twenty-eight countries were excluded due to missing data (AGO, ATG, CAF, DJI, GNQ, ERI, ETH, GAB, GRD, GIN, LBY, FSM, MOZ, NIC, MKD, PSE, PRI, SAU, SOM, SSD, VCT, SDN, SUR, SYR, TJK, TTO, VUT, and VEN) (Figure 1).

Table 1 summarizes ASDR and cardiometabolic risk factors in 2000 and 2015, along with absolute changes over the 15-year period. Between 2000 and 2015, mean ASDRs declined substantially for most cardiovascular outcomes. The mean ASDR for IHD decreased from 149.07 to 116.73 per 100,000 population (Δ −32.34), and total stroke declined from 116.59 to 90.41 (Δ −26.18). Reductions were also observed for IS (Δ −11.35) and HS (Δ −14.84). In contrast, HHD mortality remained relatively unchanged over the study period (Δ −0.01).

Regarding cardiometabolic risk factors, mean systolic blood pressure showed a slight decline (126.60 to 126.12 mmHg; Δ −0.47), and mean total cholesterol decreased modestly (4.66 to 4.56 mmol/L; Δ −0.10). In contrast, obesity prevalence increased from 0.13 to 0.20 (Δ 0.06), and diabetes prevalence increased from 0.08 to 0.11 (Δ 0.03), indicating worsening trends in metabolic risk factors despite overall declines in cardiovascular mortality.

### 3.2. Main Analysis: Associations Between Cardiometabolic Risk Factor Changes and Changes in Age-Standardized Death Rates (2000–2015)

As shown in Table 2, crude models demonstrated several significant associations between standardized changes in cardiometabolic risk factors and changes in cause-specific ASDR; however, many of these associations were attenuated after multivariable adjustment. For HHD and total stroke, no statistically significant associations were observed in either crude or adjusted analyses.

For IHD, crude analyses showed positive associations of systolic blood pressure (β = 10.66, *p* = 0.003), total cholesterol (β = 18.35, *p* < 0.001), and the composite Z-sum index (β = 3.65, *p* = 0.006) with changes in ASDR (Table 2). However, after simultaneous adjustment for other cardiometabolic risk factors, income group, and behavioral changes, only total cholesterol remained independently associated with change in IHD ASDR (adjusted β = 13.09, *p* = 0.003), whereas the composite index was no longer statistically significant (adjusted β = 2.07, *p* = 0.177). The adjusted model explained approximately 25–28% of the variance in change in ASDR.

For stroke subtypes, crude models indicated significant associations for IS, including systolic blood pressure (β = 3.71, *p* = 0.006), total cholesterol (β = 5.21, *p* < 0.001), and the composite index (β = 1.42, *p* = 0.004); however, these associations were attenuated and no longer statistically significant after adjustment. For HS, crude models showed an inverse association between systolic blood pressure change and change in ASDR (β = −3.88, *p* = 0.012), as well as a significant association for the composite index (β = −1.11, *p* = 0.048), but these findings did not persist in fully adjusted models. Overall, as shown in Table 2, among the four cardiometabolic risk factors examined, change in total cholesterol demonstrated the most consistent independent association with change in IHD ASDR, whereas other risk factor changes and the composite index did not show robust independent associations across outcomes after full adjustment.

Sensitivity analyses using non-standardized changes in cardiometabolic risk factors yielded consistent results. In these models, the association between cholesterol change and IHD mortality remained statistically significant (β = 21.16, *p* = 0.014). Cholesterol change was also significantly associated with changes in ischemic stroke mortality (β = 23.77, *p* = 0.006). These findings were directionally consistent with the main models, indicating that the results were robust to the use of z-score standardization.

### 3.3. Non-Linear Associations Based on Quartiles of Cardiometabolic Risk Factor Changes

To explore potential non-linear associations between cardiometabolic risk factor changes and changes in ASDR, we categorized country-level changes in each risk factor into quartiles (Q1–Q4) and fitted linear regression models using Q1 as the reference group (Table 3). For IHD, crude models demonstrated a graded increase in ΔASDR across quartiles of total cholesterol change. Compared with countries in the lowest quartile (Q1), those in higher quartiles showed progressively larger increases in ΔASDR (Q2: β = 36.78, *p* < 0.001; Q3: β = 43.02, *p* < 0.001; Q4: β = 51.05, *p* < 0.001). This graded association remained statistically significant after adjustment (Q2: β = 31.14, *p* = 0.004; Q3: β = 29.98, *p* = 0.006; Q4: β = 36.68, *p* = 0.002), indicating a robust relationship between greater increases in total cholesterol and greater increases in IHD ASDR. In contrast, quartiles of systolic blood pressure and body mass index did not show consistent associations after adjustment.

For diabetes, countries in the second quartile (Q2) of change showed a significant inverse association with IHD ASDR in both crude (β = −26.43, *p* = 0.009) and adjusted models (β = −20.85, *p* = 0.024), but higher quartiles did not demonstrate a monotonic pattern. For IS, crude models suggested higher ΔASDR in upper quartiles of systolic blood pressure and total cholesterol; however, these associations were attenuated and no longer statistically significant after adjustment. Diabetes change in the second quartile remained significantly associated with lower ΔASDR in adjusted analyses (β = −10.24, *p* = 0.004), without evidence of a clear dose–response pattern. Overall, as shown in Table 3, quartile-based analyses supported a graded association between total cholesterol change and IHD ASDR, whereas associations for other cardiometabolic risk factors were weaker and less consistent after adjustment.

### 3.4. Income-Stratified Associations Between Cardiometabolic Risk Factor Changes and Changes in Age-Standardized Death Rates

To examine potential heterogeneity by national income level, we conducted income-stratified analyses using World Bank income groups (Table 4). In high-income countries (n = 54), change in total cholesterol was significantly associated with change in IHD death rate in fully adjusted models (β = 16.61, *p* = 0.021), whereas changes in systolic blood pressure, obesity, and diabetes were not statistically significant. The composite metabolic change index was also not associated with IHD death rate in this group, and no significant associations were observed for IS.

In upper middle-income countries (n = 43), change in total cholesterol remained significantly associated with change in IHD death rate (β = 21.20, *p* = 0.029), and the composite metabolic change index was also significant (β = 7.64, *p* = 0.034). For IS, both total cholesterol (β = 7.04, *p* = 0.011) and the composite index (β = 2.13, *p* = 0.038) were significantly associated with change in death rate. In contrast, in lower middle-income (n = 42) and low-income countries (n = 18), no statistically significant associations were observed in adjusted models, although borderline associations were noted for systolic blood pressure (β = −20.66, *p* = 0.053) and the composite index (β = −12.71, *p* = 0.057) with IHD death rate in low-income countries. Overall, as shown in Table 4, associations between cardiometabolic risk factor changes and changes in death rates were more evident in high- and upper middle-income countries and were weaker and less consistent in lower-income settings.

## 4. Discussion

In this multinational ecological analysis of 157 countries, we examined whether country-level changes in major cardiometabolic risk factors between 2000 and 2015 were associated with concurrent changes in ASDR from HHD, IHD, total stroke, IS, and HS. During this period, mean death rates for IHD and stroke declined across most countries, whereas obesity and diabetes prevalence increased and systolic blood pressure and total cholesterol showed modest overall declines. In change–change analyses, total cholesterol was the only cardiometabolic risk factor that remained independently associated with change in IHD death rate after multivariable adjustment. Although certain non-linear associations were observed for diabetes in quartile-based analyses, and income-stratified analyses suggested associations for the composite metabolic index in upper–middle-income countries, these findings were not consistent across models or populations.

The observed association between change in total cholesterol and change in IHD death rate is consistent with extensive epidemiologic and genetic evidence supporting the well-established role of cholesterol-containing lipoproteins in the development and progression of atherosclerotic CVD [8]. Importantly, within the context of this study, changes in total cholesterol at the population level may reflect the combined effects of treatment uptake (e.g., statin use), dietary changes, and broader system-level prevention strategies, rather than a direct ecological effect of cholesterol itself. Large-scale randomized trials and collaborative meta-analyses have consistently shown that lowering low-density lipoprotein cholesterol leads to proportional reductions in major vascular events, providing strong evidence for a causal role of lipids in atherosclerotic CVD [9]. At the population level, declines in total cholesterol have occurred alongside broader improvements in cardiovascular risk management and healthcare systems in many countries since the early 2000s [10]. Total cholesterol reflects a composite of multiple lipoprotein fractions, including LDL-C and HDL-C. Because the NCD-RisC dataset provides only mean total cholesterol estimates and does not report lipoprotein subfractions separately, the present analysis cannot distinguish whether the observed changes primarily reflect shifts in LDL-C, HDL-C, or other lipid components. Countries with similar changes in total cholesterol may therefore have experienced different underlying lipoprotein trajectories. Our findings extend this evidence by suggesting that countries experiencing more favorable shifts in cholesterol levels over time also tended to experience greater reductions in IHD death rates. Importantly, however, the present study does not allow inference at the individual level. These findings should therefore be interpreted as exploratory country-level associations rather than causal relationships. The observed country-level associations may reflect the combined influence of multiple factors operating simultaneously at the population level, including pharmacologic lipid lowering, dietary changes, healthcare access, and broader health system capacity. In this context, changes in total cholesterol may also act as a proxy for broader cardiovascular prevention strategies implemented at the population level, rather than representing a direct causal pathway. Previous modeling studies have suggested that substantial declines in IHD death rates are attributable to a combination of improvements in medical therapies and reductions in major cardiovascular risk factors at the population level [11]. At the same time, because our analysis was conducted at the country level, these associations may also reflect residual confounding and contextual influences inherent to ecological analyses [12].

Although systolic blood pressure, obesity, and diabetes are well-established determinants of cardiovascular risk [13,14], their changes were not independently associated with changes in IHD death rate after full adjustment in our models. Several explanations may account for this pattern. First, cardiometabolic risk factors frequently cluster and are biologically interrelated, which may introduce multicollinearity when modeled simultaneously [15,16]. In such settings, the independent contribution of individual risk factor changes may be difficult to disentangle. Second, temporal changes in hypertension-related cardiovascular death rates suggest that the relationship between population blood pressure levels and cardiovascular death rate may have evolved over time, potentially reflecting improvements in hypertension management and broader changes in cardiovascular care [17]. As treatment intensity increases, the association between average exposure levels and cardiovascular death rate may become attenuated at the ecological level. Third, the magnitude of change in certain risk factors over the 15-year period was modest at the country level, which may have limited statistical power to detect independent associations. Finally, residual confounding by unmeasured structural determinants—including healthcare access, health system performance, medication availability (such as lipid-lowering therapies), and demographic aging—cannot be excluded and may have influenced the observed country-level associations. Although national income group was included as a covariate and may serve as a partial proxy for differences in healthcare access and system capacity across countries, it does not fully capture the complexity of these structural factors. In addition, the analytical framework used in this study may not be sufficiently sensitive to detect independent effects of interrelated cardiometabolic risk factors, particularly when these factors change concurrently and are strongly correlated at the population level.

Associations between cardiometabolic risk factor changes and stroke death rates were generally weaker and less consistent than those observed for IHD. Stroke is a heterogeneous condition encompassing both ischaemic and haemorrhagic subtypes, which differ in underlying pathophysiology and relative contributions of specific risk factors [18]. While elevated blood pressure is a major determinant of stroke incidence and is strongly associated with fatal stroke subtypes, particularly intracerebral haemorrhage [19], evidence from randomized trials has shown that organized stroke unit care significantly reduces mortality and dependency, highlighting the critical role of acute care systems in determining stroke outcomes [20]. In this context, changes in cardiometabolic risk factor levels may not translate directly into proportional changes in stroke death rates at the country level. Improvements in acute stroke care and reductions in case-fatality over time may partially decouple incidence trends from mortality trends [21]. The absence of consistent associations in adjusted models should therefore not be interpreted as evidence that these risk factors lack biological relevance, but rather that their population-level impact on death rate may be mediated by multiple additional factors not directly measured in this study.

HHD was included as an outcome because it reflects the long-term population burden of elevated blood pressure and is routinely reported in global cardiovascular mortality statistics. However, the relatively small change in HHD ASDR between 2000 and 2015 may have limited the statistical sensitivity to detect associations with cardiometabolic risk factor changes in this ecological analysis. The absence of clear associations for HHD should therefore be interpreted cautiously and may reflect limited variability in the outcome rather than a true absence of a relationship.

We additionally explored heterogeneity by national income group and observed meaningful differences across income strata. In high- and upper middle-income countries, changes in total cholesterol remained significantly associated with changes in IHD death rate, whereas such associations were not observed in lower middle- or low-income countries. In upper middle-income countries, both total cholesterol change and the composite metabolic change index were associated with changes in IHD and IS death rates, suggesting that in settings undergoing epidemiologic and health system transitions, aggregate shifts in metabolic risk profiles may be more strongly reflected in mortality patterns. By contrast, associations were weaker and less consistent in lower-middle- and low-income countries, where structural constraints, limited healthcare access, competing causes of mortality, and measurement variability may attenuate observable ecological associations. These findings suggest that the degree to which improvements in cardiometabolic risk factors translate into reductions in death rates may depend in part on underlying health system capacity and stage of epidemiologic transition.

Differences in healthcare access, preventive service coverage, pharmaceutical availability, and health system performance across income strata may influence the extent to which changes in cardiometabolic risk factors are reflected in changes in death rates [22]. In higher-income settings, greater access to preventive services, lipid-lowering medications, and organized cardiovascular care may facilitate a more direct translation of improvements in risk factor profiles into reductions in cardiovascular mortality. In contrast, in lower-income settings, limited healthcare access, lower availability of preventive services and medications, and competing health burdens may attenuate or obscure these associations at the population level. In this context, changes in total cholesterol may also act as a proxy for broader cardiovascular prevention strategies, particularly in higher-income settings where access to pharmacologic treatment, healthcare system capacity, and lifestyle interventions is more widely established, rather than representing a direct causal pathway. Structural and socioeconomic determinants of health are known to shape cardiovascular outcomes across diverse populations and health systems [23]. From a policy perspective, the findings suggest that improvements in population lipid profiles may be particularly aligned with reductions in IHD death rate, especially in higher-income settings with broader access to lipid-lowering therapies and secondary prevention. However, translating these ecological observations into policy recommendations requires caution. Comprehensive cardiovascular prevention strategies must address multiple risk factors simultaneously, including hypertension, obesity, diabetes, and behavioral determinants, as emphasized in contemporary prevention guidelines [24].

This study has several strengths. We used harmonized, publicly available multinational datasets and included a large number of countries with complete data over a 15-year period. By focusing on change–change associations rather than cross-sectional levels, we aimed to assess whether shifts in population risk factor profiles moved in parallel with shifts in death rates. The use of standardized (z-score) change metrics enabled direct comparison across risk factors measured on different scales. We also examined both continuous and quartile-based specifications, conducted income-stratified analyses, and adjusted for behavioral risk factors and income group. Several limitations should be acknowledged. First, this was an ecological study using country-level aggregated data; therefore, individual-level associations cannot be inferred, and ecological fallacy is possible. Second, the primary analysis was based on changes between two observation years (2000 and 2015). While this design captures the net change over the study period, it cannot distinguish between monotonic trends and non-monotonic trajectories over time. In addition, cardiovascular mortality reflects cumulative exposures over extended periods, and contemporaneous change–change analyses may not fully capture potential latency between changes in cardiometabolic risk factors and mortality outcomes. The use of the same time window for both exposure and outcome may therefore oversimplify temporal relationships, and the observed associations should be interpreted as reflecting overall co-variation rather than precise temporal causality. Countries may have experienced intermediate fluctuations in cardiometabolic risk factors or mortality rates that are not reflected when only the end points are compared. Future studies using continuous time-series or panel data would allow a more detailed characterization of temporal trajectories and provide additional insight into intermediate patterns. Third, residual confounding by unmeasured factors—such as medication coverage, dietary transitions, urbanization, healthcare system capacity, and demographic aging—may have influenced the observed associations. Fourth, income group was treated as time-invariant and may not reflect economic transitions during the study period. Fifth, although we used standardized estimates from international data sources, differences in modeling approaches and underlying data quality across countries may introduce measurement heterogeneity [25,26,27]. In addition, substantial heterogeneity exists across countries in terms of healthcare systems, epidemiological transitions, and risk factor measurement practices, which may influence both exposure and outcome patterns and limit the comparability of associations across settings. While income-stratified analyses partially account for this variation, they may not fully capture regional or contextual differences across countries. Future studies incorporating region-specific or stratified analyses may help to further elucidate these patterns. Sixth, the exclusion of 28 countries due to missing data—many of which were low-income or politically unstable settings—may have introduced selection bias, potentially affecting income-stratified estimates, particularly within the low-income group. Finally, absolute changes and z-score transformations reflect relative change across countries and should not be interpreted as absolute clinical risk thresholds. Although this approach improves comparability across risk factors measured on different scales, the resulting effect sizes cannot be interpreted in clinically meaningful units and may also inflate the apparent relative importance of variables with smaller variance, thereby limiting their translational relevance.

## 5. Conclusions

In this multinational ecological study of 157 countries, changes in total cholesterol demonstrated the most consistent association with changes in IHD death rate between 2000 and 2015. In the overall analysis, changes in systolic blood pressure, obesity, diabetes, and the composite metabolic index were not consistently independently associated after multivariable adjustment. However, income-stratified analyses indicated significant associations for the composite index in upper-middle-income countries, and non-linear analyses suggested an association for diabetes within specific quartiles. Associations with stroke outcomes were generally weaker and less consistent. Differences observed across income groups suggest that the extent to which improvements in cardiometabolic risk factors translate into mortality reductions may depend on broader health system and socioeconomic contexts.

Given the ecological design, these findings should not be interpreted as evidence of individual-level causality. Nonetheless, the results are best interpreted as reflecting population-level co-variation between concurrent changes in lipid profiles and IHD mortality at the country level, particularly in higher-income settings. Continued efforts to address multiple cardiometabolic risk factors in an integrated manner remain essential. Further longitudinal and individual-level research is needed to clarify how population risk factor dynamics interact with health system capacity to influence cardiovascular mortality.

## Figures and Tables

**Table 1:** Age-standardized death rates (ASDR) and cardiometabolic risk factors in 2000 and 2015, and absolute changes from 2000 to 2015 among countries included in the main analysis (n = 157).

Variable	2000, Mean (SD)	2015, Mean (SD)	Δ (2015–2000), Mean (SD)
Outcome (ASDR)			
Hypertensive heart disease	22.70 (18.06)	22.69 (17.18)	−0.01 (10.22)
Ischaemic heart disease	149.07 (93.69)	116.73 (70.84)	−32.34 (45.80)
Total stroke	116.59 (57.66)	90.41 (53.10)	−26.18 (31.08)
Ischaemic stroke	55.61 (27.37)	44.26 (24.29)	−11.35 (16.98)
Haemorrhagic stroke	60.99 (42.14)	46.15 (36.17)	−14.84 (19.35)
Risk Factors			
Systolic blood pressure	126.60 (3.24)	126.12 (3.84)	−0.47 (2.61)
Obesity prevalence (BMI ≥ 30 kg/m^2^)	0.13 (0.09)	0.20 (0.11)	0.06 (0.03)
Diabetes prevalence	0.08 (0.04)	0.11 (0.05)	0.03 (0.02)
Total cholesterol	4.66 (0.52)	4.56 (0.38)	−0.10 (0.22)

Values are presented as mean (standard deviation). Δ represents the absolute change between 2000 and 2015 (2015 minus 2000). ASDR indicates age-standardized death rate per 100,000 population. Systolic blood pressure is expressed in mmHg. Obesity and diabetes are presented as prevalence (proportion of the population); their changes represent absolute differences in prevalence. Total cholesterol is expressed in mmol/L.

**Table 2:** Associations between standardized changes in cardiometabolic risk factors and changes in ASDR in crude and adjusted models.

Predictor (per 1 SD Increase)	Crude		Adjusted	
	β (95% CI)	*p*-Value	β (95% CI)	*p*-Value
**Outcome 1: Hypertensive heart disease (Δ HHD)**				
Individual models				
SBP	0.23 (−1.38, 1.84)	0.784	1.39 (−0.75, 3.53)	0.207
BMI	1.19 (−0.42, 2.80)	0.148	1.16 (−0.90, 3.22)	0.268
DM	−0.60 (−2.21, 1.01)	0.467	−1.78 (−3.86, 0.30)	0.096
CHOL	0.99 (−0.62, 2.60)	0.228	−0.51 (−2.71, 1.69)	0.649
Composite model				
Z-sum	−0.02 (−0.61, 0.57)	0.938	0.10 (−0.66, 0.86)	0.794
**Outcome 2: Ischaemic heart disease (Δ IHD)**				
Individual models				
SBP	10.66 (3.64, 17.68)	0.003	−1.41 (−9.70, 6.88)	0.739
BMI	−5.33 (−12.48, 1.82)	0.146	−2.89 (−10.81, 5.03)	0.475
DM	3.99 (−3.20, 11.18)	0.279	4.22 (−3.82, 12.26)	0.305
CHOL	18.35 (11.74, 24.96)	<0.001	13.09 (4.58, 21.60)	0.003
Composite model				
Z-sum	3.65 (1.10, 6.20)	0.006	2.07 (−0.93, 5.07)	0.177
**Outcome 3: Total stroke (Δ STROKE)**				
Individual models				
SBP	−0.16 (−5.06, 4.74)	0.948	−4.95 (−11.48, 1.58)	0.139
BMI	−0.79 (−5.69, 4.11)	0.751	1.53 (−4.70, 7.76)	0.632
DM	0.94 (−3.96, 5.84)	0.706	1.98 (−4.35, 8.31)	0.541
CHOL	2.32 (−2.56, 7.20)	0.354	1.44 (−5.24, 8.12)	0.673
Composite model				
Z-sum	0.30 (−1.48, 2.08)	0.738	0.08 (−2.23, 2.39)	0.947
**Outcome 4: Ischaemic stroke (Δ IS)**				
Individual models				
SBP	3.71 (1.10, 6.32)	0.006	−1.60 (−4.89, 1.69)	0.345
BMI	−0.31 (−2.98, 2.36)	0.820	0.32 (−2.84, 3.48)	0.844
DM	2.11 (−0.54, 4.76)	0.122	2.32 (−0.87, 5.51)	0.157
CHOL	5.21 (2.66, 7.76)	<0.001	2.46 (−0.93, 5.85)	0.157
Composite model				
Z-sum	1.42 (0.48, 2.36)	0.004	0.78 (−0.40, 1.96)	0.197
**Outcome 5: Haemorrhagic stroke (Δ HS)**				
Individual models				
SBP	−3.88 (−6.86, −0.90)	0.012	−3.36 (−7.40, 0.68)	0.105
BMI	−0.48 (−3.52, 2.56)	0.756	1.21 (−2.63, 5.05)	0.539
DM	−1.16 (−4.20, 1.88)	0.455	−0.35 (−4.25, 3.55)	0.862
CHOL	−2.90 (−5.92, 0.12)	0.062	−1.02 (−5.16, 3.12)	0.631
Composite model				
Z-sum	−1.11 (−2.21, −0.01)	0.048	−0.70 (−2.13, 0.73)	0.341

Values are β coefficients (95% confidence intervals) from linear regression models. Crude models represent univariable associations between each standardized risk factor change and change in age-standardized death rate (ΔASDR). Adjusted models were fitted including all four standardized cardiometabolic risk factor changes simultaneously and were additionally adjusted for World Bank income group (reference: high income) and absolute changes in smoking prevalence, alcohol consumption, and insufficient physical activity between 2000 and 2015. Risk factor changes were standardized as Z-scores (mean = 0, SD = 1). Thus, β represents the change in ΔASDR (deaths per 100,000 population) associated with a 1-SD increase in the change of each risk factor. Δ indicates absolute change (2015–2000). n = 157 countries.

**Table 3:** Associations of quartiles of cardiometabolic risk factor changes with changes in ASDR: crude and adjusted linear regression models for IHD and IS.

Risk Factor (Quartiles of Change)	Crude		Adjusted	
	β (95% CI)	*p*-Value	β (95% CI)	*p*-Value
Outcome 1: Ischaemic heart disease (Δ IHD)				
SBP				
Q1 (Reference)	Ref.	-	Ref.	-
Q2	23.00 (3.22, 42.78)	0.024	14.28 (−5.54, 34.10)	0.160
Q3	24.68 (4.90, 44.46)	0.016	19.28 (−3.40, 41.96)	0.098
Q4	28.14 (8.36, 47.92)	0.006	8.37 (−12.88, 29.62)	0.441
BMI				
Q1 (Reference)	Ref.	-	Ref.	-
Q2	−1.47 (−21.80, 18.86)	0.888	5.25 (−12.82, 23.32)	0.570
Q3	−6.61 (−26.94, 13.72)	0.525	−5.28 (−23.94, 13.38)	0.580
Q4	−10.24 (−30.57, 10.09)	0.325	6.33 (−13.41, 26.07)	0.530
DM				
Q1 (Reference)	Ref.	-	Ref.	-
Q2	−26.43 (−46.01, −6.85)	0.009	−20.85 (−38.82, −2.88)	0.024
Q3	8.30 (−11.28, 27.88)	0.407	6.38 (−12.14, 24.90)	0.501
Q4	−7.65 (−27.23, 11.93)	0.445	−9.35 (−29.26, 10.56)	0.359
CHOL				
Q1 (Reference)	Ref.	-	Ref.	-
Q2	36.78 (18.38, 55.18)	<0.001	31.14 (10.48, 51.80)	0.004
Q3	43.02 (24.62, 61.42)	<0.001	29.98 (8.75, 51.21)	0.006
Q4	51.05 (32.65, 69.45)	<0.001	36.68 (14.12, 59.24)	0.002
Outcome 2: Ischaemic stroke (Δ IS)				
SBP				
Q1 (Reference)	Ref.	-	Ref.	-
Q2	7.48 (0.13, 14.83)	0.048	0.77 (−7.01, 8.55)	0.846
Q3	10.81 (3.46, 18.16)	0.005	2.61 (−6.31, 11.53)	0.567
Q4	7.40 (0.05, 14.75)	0.050	−2.54 (−10.89, 5.81)	0.552
BMI				
Q1 (Reference)	Ref.	-	Ref.	-
Q2	−2.18 (−9.69, 5.33)	0.570	−0.91 (−7.97, 6.15)	0.802
Q3	2.86 (−4.65, 10.37)	0.457	3.48 (−3.81, 10.77)	0.351
Q4	−1.36 (−8.87, 6.15)	0.724	3.25 (−4.47, 10.97)	0.410
DM				
Q1 (Reference)	Ref.	-	Ref.	-
Q2	−9.74 (−16.99, −2.49)	0.009	−10.24 (−17.18, −3.30)	0.004
Q3	2.83 (−4.42, 10.08)	0.446	1.76 (−5.39, 8.91)	0.631
Q4	−0.35 (−7.60, 6.90)	0.925	−0.07 (−7.75, 7.61)	0.985
CHOL				
Q1 (Reference)	Ref.	-	Ref.	-
Q2	7.54 (0.35, 14.73)	0.042	3.85 (−4.50, 12.20)	0.368
Q3	9.58 (2.39, 16.77)	0.010	2.60 (−5.97, 11.17)	0.553
Q4	14.41 (7.22, 21.60)	<0.001	6.08 (−3.03, 15.19)	0.192

β values represent regression coefficients (95% confidence intervals) from linear regression models. Quartiles were defined according to the distribution of absolute changes in each risk factor between 2000 and 2015, with Q1 (lowest change) as the reference category. Adjusted models included World Bank income group (reference: high income) and absolute changes in smoking prevalence, alcohol consumption, and insufficient physical activity as covariates. Overall *p*-values were obtained from analysis of variance comparing models with and without the quartile exposure variable (global test for the quartile effect). Δ indicates absolute change (2015–2000). ASDR indicates age-standardized death rate per 100,000 population.

**Table 4:** Income-stratified associations of standardized cardiometabolic risk factor changes with changes in ASDR.

Outcome	β (95% CI)	*p*-Value
High income (n = 54)		
Ischaemic heart disease (Δ IHD)		
SBP	7.01 (−7.12, 21.14)	0.336
BMI	−5.86 (−19.80, 8.08)	0.414
DM	−7.07 (−23.22, 9.08)	0.395
CHOL	16.61 (3.05, 30.17)	0.021
Z-sum	0.10 (−4.66, 4.86)	0.966
Ischaemic stroke (Δ IS)		
SBP	0.36 (−6.46, 7.18)	0.918
BMI	2.84 (−3.88, 9.56)	0.412
DM	−0.03 (−7.83, 7.77)	0.994
CHOL	0.30 (−6.25, 6.85)	0.930
Z-sum	1.07 (−1.03, 3.17)	0.324
Upper middle income (n = 43)		
Ischaemic heart disease (Δ IHD)		
SBP	−14.96 (−36.64, 6.72)	0.185
BMI	5.76 (−13.25, 24.77)	0.556
DM	12.38 (−4.93, 29.69)	0.169
CHOL	21.20 (2.97, 39.43)	0.029
Z-sum	7.64 (0.82, 14.46)	0.034
Ischaemic stroke (Δ IS)		
SBP	−3.57 (−9.69, 2.55)	0.259
BMI	3.11 (−2.24, 8.46)	0.264
DM	1.00 (−3.88, 5.88)	0.691
CHOL	7.04 (1.90, 12.18)	0.011
Z-sum	2.13 (0.19, 4.07)	0.038
Lower middle income (n = 42)		
Ischaemic heart disease (Δ IHD)		
SBP	8.22 (−6.19, 22.63)	0.272
BMI	−10.15 (−24.83, 4.53)	0.184
DM	5.62 (−7.22, 18.46)	0.397
CHOL	−8.47 (−25.60, 8.66)	0.339
Z-sum	0.42 (−6.03, 6.87)	0.898
Ischaemic stroke (Δ IS)		
SBP	0.58 (−6.36, 7.52)	0.871
BMI	−6.28 (−13.34, 0.78)	0.090
DM	3.16 (−3.01, 9.33)	0.324
CHOL	−0.90 (−9.13, 7.33)	0.832
Z-sum	−0.69 (−3.79, 2.41)	0.665
Low income (n = 18)		
Ischaemic heart disease (Δ IHD)		
SBP	−20.66 (−39.16, −2.16)	0.053
BMI	3.85 (−23.20, 30.90)	0.786
DM	−14.31 (−50.08, 21.46)	0.451
CHOL	34.33 (−20.66, 89.32)	0.249
Z-sum	−12.71 (−24.61, −0.81)	0.057
Ischaemic stroke (Δ IS)		
SBP	−7.37 (−16.78, 2.04)	0.156
BMI	7.25 (−6.51, 21.01)	0.326
DM	−4.54 (−22.73, 13.65)	0.635
CHOL	19.78 (−8.19, 47.75)	0.196
Z-sum	−2.28 (−8.43, 3.87)	0.480

Values are β coefficients (95% confidence intervals) from multivariable linear regression models stratified by World Bank income group. Changes in cardiometabolic risk factors (2015–2000) were standardized as z-scores (mean = 0, SD = 1). Models were adjusted for absolute changes in smoking prevalence, alcohol consumption, and insufficient physical activity. Overall *p*-values were obtained by comparing models with and without the exposure term(s) (global test). ASDR indicates age-standardized death rate per 100,000 population. Δ indicates absolute change (2015–2000).

## Data Availability

The raw data supporting the conclusions of this article will be made available by the authors on request.
